# *Ehrlichia chaffeensis* DNA in *Haemaphysalis longicornis* Ticks, Connecticut, USA

**DOI:** 10.3201/eid3106.250034

**Published:** 2025-06

**Authors:** Goudarz Molaei, Amrita Ray Mohapatra, Noelle Khalil, Duncan Cozens, Denise Bonilla

**Affiliations:** The Connecticut Agricultural Experiment Station, New Haven, Connecticut, USA (G. Molaei, A.R. Mohapatra, N. Khalil, D. Cozens); US Department of Agriculture Animal and Plant Health Inspection Service, Fort Collins, Colorado, USA (D. Bonilla)

**Keywords:** *Ehrlichia chaffeensis*, *Haemaphysalis longicornis*, ticks, tickborne diseases, vector-borne infections, bacteria, Connecticut, United States

## Abstract

Informed by passive tick surveillance, we collected questing *Haemaphysalis longicornis* ticks from southwestern Connecticut, USA. Of 445 ticks tested by PCR, 3 nymphs were positive: 1 for *Ehrlichia chaffeensis* and 2 for *Borrelia burgdorferi*. This finding highlights the enduring public health challenges of invasive ticks and associated pathogens.

*Ehrlichia chaffeensis* is the most common causative agent of human monocytic ehrlichiosis (HME) and is transmitted primarily by the lone star tick (*Amblyomma americanum*) (*1*). Frequently reported from the southeast and south central United States, HME cases increased nearly 15-fold during 2001–2019 (from 142 to 2,093 cases), and then decreased substantially in 2020 (n = 1,178 cases), likely due to the COVID-19 pandemic. In subsequent years, disease cases remained lower than prepandemic levels. In Connecticut, reported HME cases totaled just 2 during 2008–2018; however, since 2019, reports from Connecticut indicated an annual recurrence of the disease, and cases increased to a total of 28 during 2019–2023. As with other tickborne diseases, convincing evidence indicates the number of HME cases is underreported and because of the recent range expansion of *A. americanum*, particularly in northeast sections of the United States, investigators anticipate an increase in disease cases (*2*).

Native to eastern Asia and invasive to Australia, New Zealand, and several Pacific Islands, the first report of *Haemaphysalis longicornis* in the United States came from New Jersey in 2017 (*3*), and the species subsequently spread into at least 21 mostly eastern and northeastern states (Figure, panel A) (*4*). Because of its wide host range and ability to survive in an expansive breadth of climatic conditions, *H. longicornis* will likely spread to and establish populations across a large portion of the United States (*5*). This tick is a known vector of a wide array of pathogens in its native and invasive ranges, and researchers have detected genetic materials from *Anaplasma phagocytophilum*, *Babesia microti*, *Borrelia burgdorferi*, Bourbon virus, and *Theileria orientalis* Ikeda in environmentally collected specimens in the United States; however, its vector potential for many of these pathogens remains unclear (*6*–*9*). We screened ticks collected in Connecticut to assess potential human pathogens.

**Figure:**
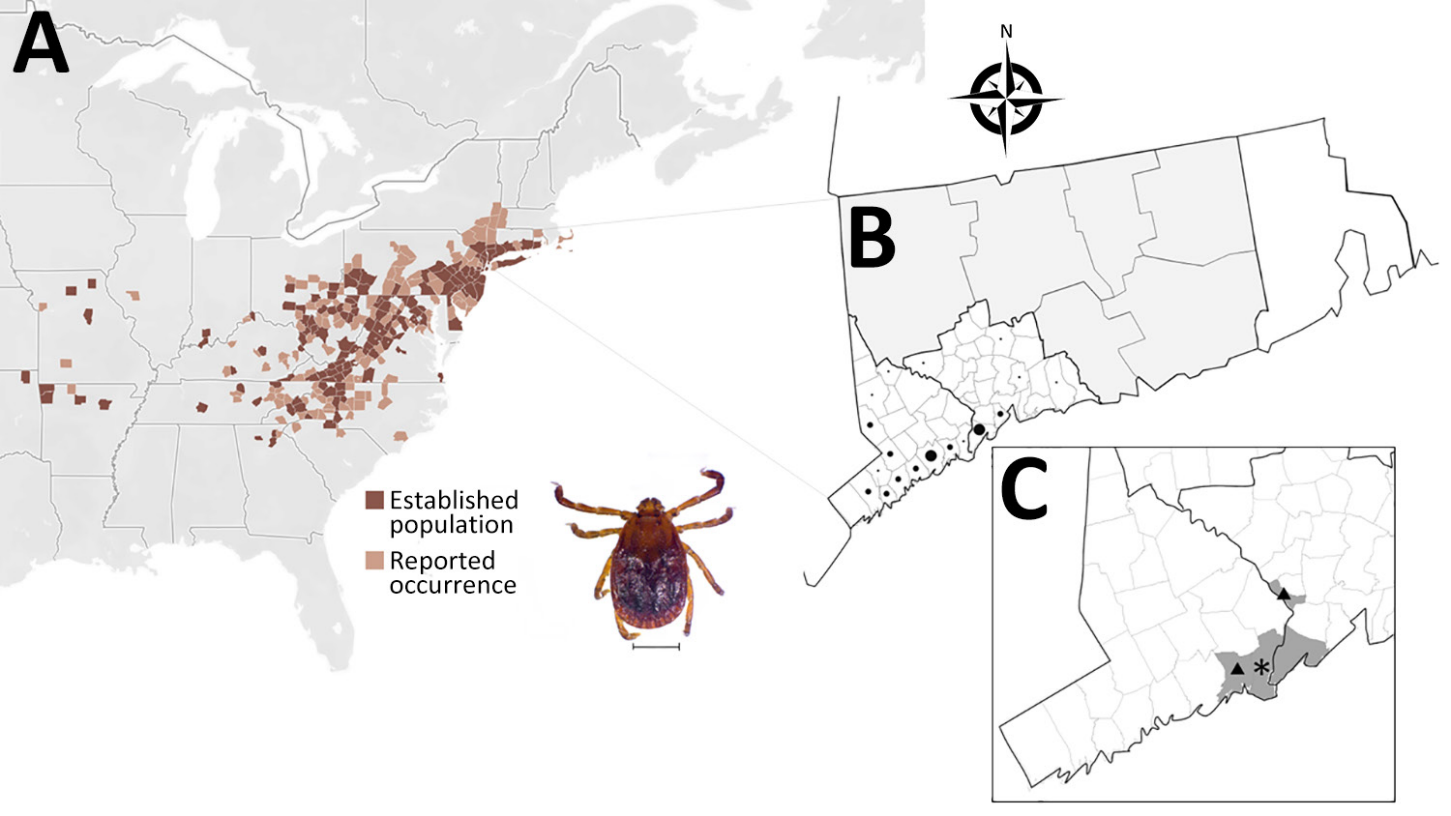
Maps showing range and locations of ticks in a study of *Ehrlichia chaffeensis* DNA in *Haemaphysalis longicornis* ticks, Connecticut, USA. A) Map of eastern United States showing states with established populations or reported occurrence of *H. longicornis***.** B) For comparison, map of Connecticut showing locations (dots) where residents reported removing *H. longicornis* ticks that they submitted to the Connecticut Agricultural Experiment Station Tick Testing Laboratory. This area overlaps with area in which *E. chaffeensis*–positive *H. longicornis* ticks were collected for this study (C); dot size indicates number of ticks collected per area, either 1, 2–5, or >5 ticks. C) Map of southwestern part of the state showing areas of known established *H. longicornis* tick populations (shaded in gray), location of tick specimen found to be positive for *E. chaffeensis* (asterisk), and location of specimens found to be positive for *Borrelia burgdorferi* (triangles). Inset shows adult female *H. longicornis* tick; scale bar indicates 1 mm.

Of 8,700 *H. longicornis* larvae (n = 8,120), nymphs (n = 412), and adult female ticks (n = 168) we collected from 4 towns in southwestern Connecticut during 2021–2024, we tested 88 females and 357 nymphs for evidence of infection. Of those ticks, 2 (0.6%) nymphs tested positive for *B. burgdorferi*, 1 collected in April 2021 from Bridgeport (41.159°N, 73.202°W) and 1 collected in August 2023 from Derby (41.336°N, 73.1006°W) (Appendix). In screening a subset of *H. longicornis* nymphs (n = 126), 1 (0.8%) nymph collected in May 2021 from Stratford (41.1526°N, 73.1471°W) tested positive for *E. chaffeensis* (Appendix Table). The 16S rRNA gene fragment for *E. chaffeensis* (GenBank accession no. PQ569094) from this assay showed 99.9% identity to several sequences of the same gene in the GenBank database. The cytochrome *c* oxidase subunit 1 gene fragment of the *H. longicornis* specimen (GenBank accession no. PQ561597) showed 99.7% identity to similar gene sequences in GenBank, confirming the species identity. 

The overall 0.8% *E. chaffeensis* infection rate in *H. longicornis* is similar to that in the principal vector of this pathogen, *A. americanum*, in Connecticut (1%) and substantially lower than that in the United States (5%–15%). The detection of *B. burgdorferi* in 2 *H. longicornis* nymphs with an infection rate of 0.6% is slightly higher than that reported in a study of field collections of this tick in Pennsylvania (0.4%) (*8*).

The Stratford site where the *E. chaffeensis*–positive specimen was collected is frequented by white-tailed deer, and repeated surveys have revealed that the area is heavily infested with *H. longicornis* and *A. americanum*. Both tick species are 3-host ticks (*2*,*9*), and all life stages readily feed on white-tailed deer. White-tailed deer are known reservoir hosts for *E. chaffeensis* (*1*), and have an infection rate ranging 7%–54% (*10*). Records of human *H. longicornis* bites exist in the United States (*9*), but how frequently this species will infest humans remains unclear. Evidence has also been reported on partial blood feeding in host-seeking *H. longicornis*, which could lead to pathogen transmission as the tick attempts to complete a blood meal after partially feeding on an infected host in the same life stage (*8*). *H. longicornis* could thus conceivably acquire *E. chaffeensis* directly from an infected white-tailed deer or during cofeeding with an infected *A. americanum* and transmit to humans during an initial blood meal or a secondary partial blood meal.

Aided by frequent intercontinental movement of humans and importation of animals and agricultural products, the United States has recently witnessed an increase in the introduction of invasive ticks capable of transmitting a diverse group of pathogens of public health concern. Those nonnative tick species have the potential to establish populations and expand their range under conducive climatic conditions. Thus, mitigating public and animal health risks depends on increasing public awareness of the risks associated with invasive ticks and pathogens, expanding passive and active surveillance programs, and continued diligent inspection of animals and plants. Improving the capacity to accurately identify tick species and test for native and nonnative pathogens should be an integral part of any comprehensive program designed to expand our understanding of the distribution and prevalence of tickborne infections.

Appendix Additional information for *Ehrlichia chaffeensis* DNA in *Haemaphysalis longicornis* ticks, Connecticut, USA.
