# Supplementary material for: Ehrlichia chaffeensis DNA in Haemaphysalis longicornis Ticks, Connecticut, USA
Source: Emerg Infect Dis. 2025 Jun;31(6):1260–2. doi: 10.3201/eid3106.250034 (PMC12123906; doi:10.3201/eid3106.250034)
Supplement: Appendix — Additional information for Ehrlichia chaffeensis DNA in Haemaphysalis longicornis ticks, Connecticut, USA. [file 25-0034-Techapp-s1.pdf]

Article DOI: http://doi.org/10.3201/eid3106.250034

EID cannot ensure accessibility for Supplemental Materials supplied by authors. Readers who have difficulty accessing supplementary content should contact the authors for assistance.

Ehrlichia chaffeensis DNA in *Haemaphysalis longicornis* Ticks, Connecticut, USA

Appendix

Additional Methods

Informed by the Passive Tick and Tick-borne Pathogen Surveillance Program (also known as the Connecticut Agricultural Experiment Station-Tick Testing Laboratory [CAES-TTL]) and following a submission of a human-biting *Haemaphysalis longicorni*s in 2018, frequent tick surveys have since been conducted in the state. These surveys have been performed during March–November in the towns of Bridgeport and Stratford in Fairfield County and Milford and Derby in New Haven County, areas in southwestern Connecticut with known established populations of *H. longicorni*s (Figure) (*1*). Sampling sites primarily included public recreation areas located on the Long Island Sound, a strip of shoreline located at the tip of a small peninsular near the mouth of the Housatonic River, a state park on the east bank of the Housatonic River, and a vegetated landfill with poor habitat quality, dominated by mostly invasive plant species (*1*,*2*). Questing ticks were collected by dragging a 1m^2 white felt or cotton cloth across the top of the vegetation and leaf litter (*1*). Dragging cloths were examined every 10–15 min, and ticks were removed with a pair of forceps, transferred into vials containing 75% ethanol, and transported to the CAES-TTL. Ticks were identified to species using morphological keys and were corroborated by genetic analyses targeting the *cytochrome c oxidase subunit* 1 (*COX1*) gene (*3*) for a subsample of the specimens, including the one tested positive for *Ehrlichia chaffeensis*. All collected ticks were stored at −80°C until they were used for pathogen screening and other experiments.

Tick surveys consisted of 26 events from 4 towns in southwestern Connecticut (Stratford = 5, Bridgeport = 8, Milford = 6, and Derby = 7) in April 2021–October 2024. Of the 8,700 *H. longicornis* larvae (n = 8,120), nymphs (n = 412), and adult females (n = 168) collected, 88 females and 357 nymphs were tested for evidence of infection. DNA was prepared from individual *H. longicornis* adult female and nymph homogenates on the KingFisher Flex Purification System with the MagMAX Viral/Pathogen Nucleic Acid Isolation Kit (ThermoFisher Scientific, https://www.thermofisher.com)

The extracted nucleic acids (DNA and RNA) were subjected to a multiplex real-time reverse transcription PCR assay using a Bio-Rad C1000 with a CFX96 optical module (Bio-Rad Laboratories, https://www.bio-rad.com). This assay was used to test ticks for 5 common pathogens in the Northeast: *Anaplasma phagocytophilum*, *Babesia microti, Borrelia burgdorferi, Borrelia miyamotoi*, and the lineage II strain of Powassan virus (Appendix Table) (*4*). A subsample of *H. longicornis* nymphs (n = 126) was additionally screened for evidence of Anaplasmataceae (including *Ehrlichia* and *Anaplasma*) using universal primers targeting a 1100-nt portion of the *16S rRNA* gene in a conventional PCR assay and based on established protocols (*1*,*5*). PCR amplicons were purified using a QIAquick PCR purification kit (QIAGEN, https://www.qiagen.com) and submitted for sequencing at the Keck DNA Sequencing Facility at Yale University (New Haven, CT). Sequences were then annotated using ChromasPro version 2.1.8 (Technelysium, https://technelysium.com.au) and compared with those available in the NCBI GenBank.

References

1. Molaei G, Little EAH, Williams SC, Stafford KC III. First record of established populations of the invasive pathogen vector and ectoparasite *Haemaphysalis longicornis* (Acari: Ixodidae) in Connecticut, United States. J Med Entomol. 2021;58:2508–13. PubMed https://doi.org/10.1093/jme/tjab117

2. Molaei G, Little EAH, Williams SC, Stafford KC. Bracing for the worst — range expansion of the lone star tick in the Northeastern United States. N Engl J Med. 2019;381:2189–92. PubMed https://doi.org/10.1056/NEJMp1911661

3. Folmer O, Black M, Hoeh W, Lutz R, Vrijenhoek R. DNA primers for amplification of mitochondrial c*ytochrome c oxidase* subunit I from diverse metazoan invertebrates. Mol Mar Biol Biotechnol. 1994;3:294–9. PubMed

4. Tokarz R, Tagliafierro T, Cucura DM, Rochlin I, Sameroff S, Lipkin WI. Detection of *Anaplasma phagocytophilum*, *Babesia microti*, *Borrelia burgdorferi*, *Borrelia miyamotoi*, and Powassan virus in ticks by a multiplex real-time reverse transcription-PCR assay. MSphere. 2017;2: e00151-17. PubMed https://doi.org/10.1128/mSphere.00151-17

5. Alam S, Khan M, Alouffi A, Almutairi MM, Ullah S, Numan M, et al. Spatio-temporal patterns of ticks and molecular survey of *Anaplasma marginale*, with notes on their phylogeny. Microorganisms. 2022;10:1663. PubMed https://doi.org/10.3390/microorganisms10081663

Appendix Table. A subset of adult female and nymph *Haemaphysalis longicornis* ticks collected from Stratford and Bridgeport (Fairfield County, Connecticut) and Milford and Derby (New Haven County, Connecticut) and tested for 6 common tickborne pathogens*

			Positive test results by pathogen					
Town	Adult female	Nymph	*Borrelia burgdorferi*	*Borrelia miyamotoi*	*Anaplasma phagocytophilum*	*Babesia microti*	Powassan virus	*Ehrlichia chaffeensis*
			ospA (4)	*flaB (4)*	*16S rRNA (4)*	*COX1 (4)*	*3′-UTR (4)*	*16S rRNA (5)*
Bridgeport	7	184	1	0	0	0	0	0
Stratford	6	79	0	0	0	0	0	1
Derby	68	94	1	0	0	0	0	0
Milford	7	0	0	0	0	0	0	0
Total	88	357	2	0	0	0	0	1

* ospA, outer surface protein A; flaB, flagellin B; COX1, cytochrome c oxidase; 3′-UTR, 3′-untranslated region; 16S rRNA, 16S ribosomal RNA.
